# Targeted Deletion of the First Intron of the *Wx*^*b*^ Allele via CRISPR/Cas9 Significantly Increases Grain Amylose Content in Rice

**DOI:** 10.1186/s12284-021-00548-y

**Published:** 2022-01-04

**Authors:** Xingdan Liu, Qi Ding, Wenshu Wang, Yanling Pan, Chao Tan, Yingbo Qiu, Ya Chen, Hongjing Li, Yinlong Li, Naizhong Ye, Nian Xu, Xiao Wu, Rongjian Ye, Jianfeng Liu, Chonglie Ma

**Affiliations:** 1 College of Agronomy, Hunan Agricultural University, Changsha, 410128 China; 2 Life Science and Technology Center, China National Seed Group Co., LTD, Wuhan, 430206 Hubei China; 3 State Key Laboratory of Crop Breeding Technology Innovation and Integration, China National Seed Group Co., LTD, Wuhan, 430206 Hubei China

**Keywords:** *Wx* gene, GBSSI, CRISPR/Cas9, Intron, Amylose content, Rice

## Abstract

**Background:**

The rice *Waxy* (*Wx*) gene plays a major role in seed amylose synthesis and consequently controls grain amylose content. *Wx* gene expression is highly regulated at the post-transcriptional level. In particular, the GT/TT polymorphism at the 5′splicing site of its 1st intron greatly affects this intron’s splicing efficiency and defines two predominant *Wx* alleles, *Wx*^*a*^ and *Wx*^*b*^. *Wx*^*a*^ rice often harbours intermediate to high amylose contents, whereas *Wx*^*b*^ rice exhibits low to intermediate amylose contents. By deleting the *Wx* 1st intron using CRISPR/Cas9 technology, we generate a completely novel *Wx* allele and further investigate how intron removal affects *Wx* gene expression and rice grain amylose content.

**Results:**

CRISPR/Cas9-mediated targeted deletion of the *Wx* 1st intron was performed on 4 rice inbred lines: KY131 (*Wx*^*b*^), X32 (*Wx*^*b*^), X35 (*Wx*^*a*^) and X55 (*Wx*^*lv*^). Deletion of the 1st intron occurred in 8.6–11.8% of the primary transformants of these 4 inbred lines. Compared to wild-type plants, amylose content was significantly increased from 13.0% to approximately 24.0% in KY131 and X32 mutant lines, which both carried the *Wx*^*b*^ allele. However, no significant difference in amylose content was observed between wild-type plants and X35 and X55 mutant lines, which carried the *Wx*^*a*^ and *Wx*^*lv*^ alleles, respectively. *Wx* gene expression analysis of wild-type plants and mutants yielded results that were highly consistent with amylose content results. KY131 and X32 mutants accumulated increased levels of steady mRNA transcripts compared with wild-type plants, whereas steady mRNA levels were not altered in X35 and X55 mutants compared with wild-type plants. Grain quality, including appearance quality and eating and cooking quality, which are tightly associated with amylose content, was also assessed in wild-type and mutant plants, and data were presented and analysed.

**Conclusions:**

This study presents a novel and rapid strategy to increase amylose content in inbred rice carrying a *Wx*^*b*^ allele. Our data strongly suggest that the 1st intron of the *Wx* gene regulates *Wx* gene expression mainly at the post-transcriptional level in rice. This finding is in contrast to a previous hypothesis suggesting that it influences *Wx* gene transcription. In addition, removal of the first intron generates a completely novel *Wx* allele. Further studies on this new *Wx* allele will provide invaluable insights into the regulation of *Wx* gene expression, which will help researchers engineer new *Wx* alleles to facilitate the breeding of rice cultivars with better eating and cooking quality.

**Supplementary Information:**

The online version contains supplementary material available at 10.1186/s12284-021-00548-y.

## Background

Improving grain quality is one of the most important goals in rice (*Oryza sativa* L.) breeding programs. Rice quality refers to the basic characteristics of rice in commodity circulation and includes four main aspects: milling quality, appearance quality, cooking and eating quality (ECQ) and nutritional quality. Among them, appearance quality, ECQ are particularly important (Lau et al. 2015). ECQ is determined from amylose content (AC), gel consistency (GC), gelatinization temperature (GT), and viscosity (Phing Lau et al. 2016; Tian et al. 2009). Starch accounts for up to 90% of the dry weight in rice grains (Zhou et al. 2002). Amylose, a constituent of starch, is a major indicator of ECQ in rice (Fitzgerald et al. 2009; Li et al. 2016; Tian et al. 2009). Rice is divided into four types based on amylose content: glutinous (AC < 2%), soft, intermediate to low and high (AC > 25%) (Pandey et al. 2012). Generally, the higher the amylose content in rice grain is, the less sticky and harder the cooked rice is, resulting in poor taste (Jobling 2004; Juliano 1992). However, rice with too low an amylose content is too sticky and soft. Thus, rice with intermediate to low amylose content is more popular.

The *Waxy* (*Wx*) gene (LOC_Os06g04200) encodes granule-bound starch synthase I (GBSSI), which was cloned by Wang et al. in 1990 (Wang et al. 1990). The *Wx* gene is a major gene controlling amylose content in rice endosperm and plays a decisive role in rice ECQ (Tian et al. 2009). At present, the amylose content among cultivated rice varieties is highly diversified. Such vast differences are mainly attributed to *Wx* gene allele variation. At least 9 *Wx* natural alleles have thus far been discovered and identified in rice. These alleles include *Wx*^*a*^, *Wx*^*b*^, *Wx*^*in*^, *Wx*^*op*^*/Wx*^*hp*^, *Wx*^*mq*^, *Wx*^*mp*^, *Wx*^*lv*^, *Wx*^*la*^*/Wx*^*mw*^, and *wx* (Chen et al. 2008; Dobo et al. 2010; Mikami et al. 2008; Larkin and Park 2003; Teng et al. 2012; Zhang et al. 2021, 2019; Zhou et al. 2021). DNA sequence differences between *Wx* alleles modulate *Wx* gene expression or enzyme activity, consequently causing variation in AC and grain quality. Researchers have designed different strategies to improve rice quality, including both transgenic approaches and marker-assisted selection (MAS) breeding (Jin et al. 2010; Kimiko et al. 2003; Liu et al. 2005; Phing Lau et al. 2016; Terada et al., 2000; Yu et al. 2009). In particular, introducing *Wx*^*b*^ (AC ~ 16%) and *Wx*^*in*^ (AC ~ 20%) alleles into rice germplasm with high amylose content via MAS or traditional breeding greatly expedites rice quality improvement.

With the development of rice consumption specialization, the demand for amylose content in rice is increasingly diversified. Thus, new methods to modulate amylose content or create new *Wx* alleles are needed. Third-generation genome editing technology, CRISPR/Cas9 genome editing technology, first emerged approximately a decade ago (Jinek et al. 2012; Richter et al. 2012). Since then, it has rapidly evolved into a collection of diverse genome editing tools, and these genome editing tools have been widely used in the improvement of agronomic traits for various crops (Chen et al. 2019; Fiaz et al. 2019; Gao 2021). Most importantly, the T-DNA insertion site and gene editing target sites are located in different region, and homozygous edited mutants free of transgenic components can be selected through progeny separation (Zhu et al. 2020). Compared with traditional transgenic methods, CRISPR/Cas9 technology is more prone to changes in natural mutations and has more potential to be applied to production methods. Recently, CRISPR/Cas9- mediated rice *Wx* gene editing has been intensively reported. These studies can be grouped into 4 categories. First, researchers have created new glutinous rice by completely knocking out the *Wx* gene (Ma et al. 2015; Zhang et al. 2018). Second, by editing the key cis-acting elements on the *Wx* gene promoter, researchers have achieved fine-tuning of AC in the *Wx*^*a*^ or *Wx*^*b*^ background (Huang et al. 2020a; Zeng et al. 2020). Third, researchers have tried to adjust rice AC by manipulating the splicing efficiency of the *Wx* gene at the posttranscriptional level (Zeng et al. 2020). Fourth, researchers have used CRISPR/Cas9-mediated base editors to modulate rice AC by altering GBSSI enzyme activity (Huang et al. 2021; Xu et al. 2021). These studies have not only paved new avenues for rice AC improvement but also generated a variety of new *Wx* alleles and provided new insights into the regulation of rice *Wx* gene expression and the modulation of GBSSI enzyme activity. In addition, Li et al. (1995) demonstrated that the first intron of the rice *Wx* gene greatly enhances foreign gene expression in rice protoplasts. However, whether the *Wx* 1st intron exhibits gene expression enhancement similar to its native *Wx* gene remains unclear. In situ deletion of the 1st intron from its native *Wx* gene will allow us to closely investigate this issue.

In this study, we exploited CRISPR/Cas9 gene editing technology to remove the first intron of the *Wx* gene. Intron removal generated a completely novel *Wx* allele and significantly increased the amylose content in rice inbred with the *Wx*^*b*^ allele, whereas the amylose content was minimally changed in rice inbred with the *Wx*^*a*^ allele.

## Results

### CRISPR/Cas9-Mediated Deletion of the 1st Intron of *Wx*

**Fig. 1:**
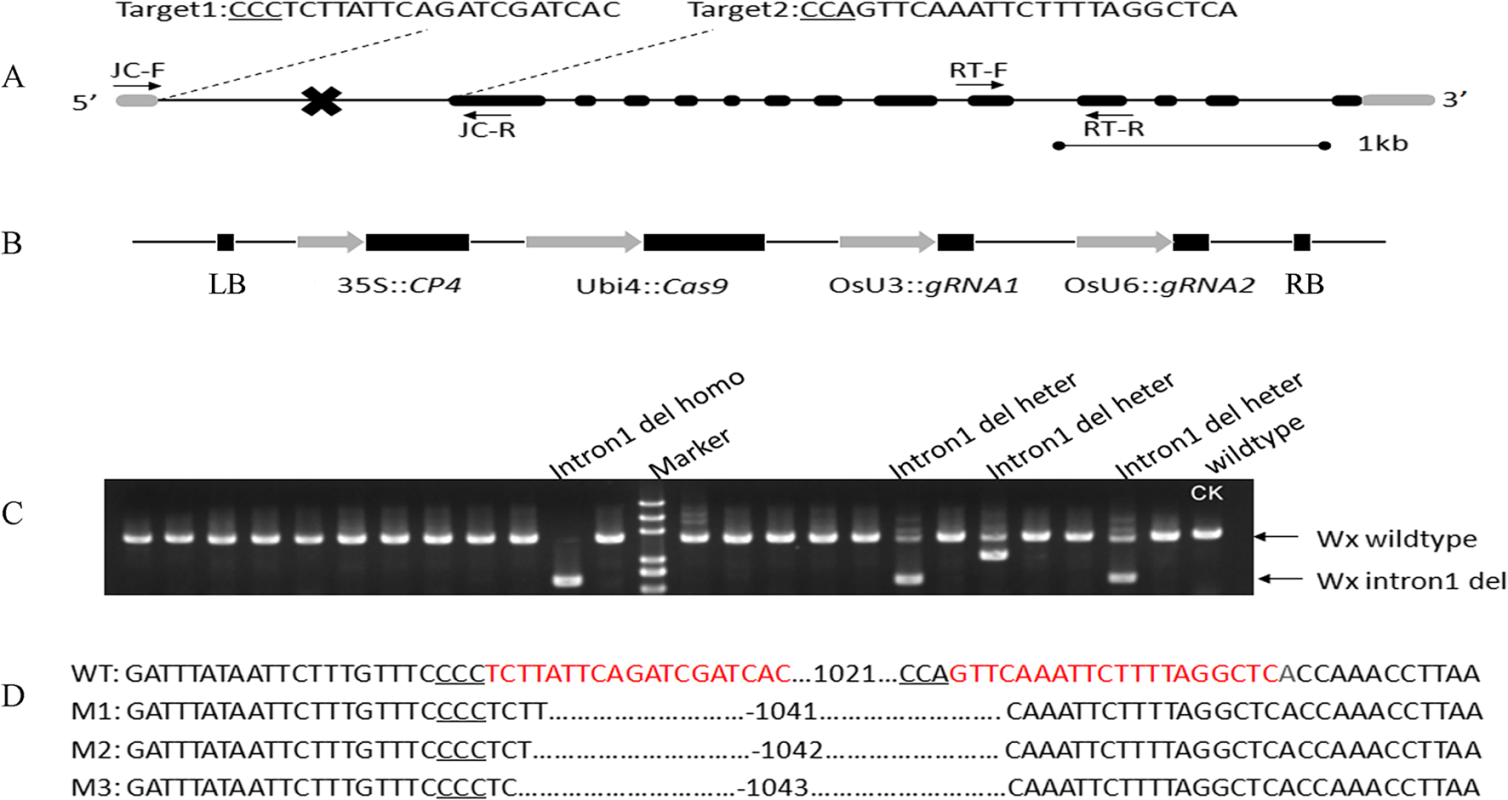
CRISPR/Ca9-mediated deletion of the *Wx* 1st intron. **A** Position and sequence of the two gRNA target sites on the genomic regions of the *Wx* gene. Introns are shown as lines. Exons are shown as boxes. PAM motifs (CCN) of two targeted sequences are underlined. Two primer pairs, JC-F/R used for the detection of large fragment deletions and RT-F/R used for *Wx* gene expression analysis via RT–qPCR, were marked at their appropriate locations. **B** Schematic diagram of the editing vector pZZT477’s T-DNA structure. LB, T-DNA left border; RB, T-DNA right border; 35S, CaMV35s promoter; CP4, *Agrobacterium tumefaciens* strain *CP4 EPSP* (5-enolpyruvylshikimate-3-phosphate synthase) gene; Ubi4, sugarcane ubiquitin *4* gene promoter; Cas9, CRISPR/Cas9 gene with codon optimized for expression in rice; U3, U6, rice U3 and U6 snRNA promoter. **C** Detection of mutations in the first intron of the *Wx* gene via PCR assay in the T0 generation. **D** Sequencing results of the first intron-deleted mutant lines. The PAM motifs are underlined, target sequences are highlighted in red, and dotted lines indicate deletions. The WT indicates the wild type, and M1, M2, and M3 are different homozygous lines in the T1 generation

To investigate how the *Wx* 1st intron regulates the amylose content of rice grains, we decided to remove the 1st intron using CRISPR/Cas9 technology. We chose four inbred rice lines with different genetic backgrounds as our testing materials. Kongyu131 (KY131) was an elite *japonica* inbred and carries a typical *Wx*^*b*^ allele. The other three X32, X35 and X55 lines were *indica* lines inbred from our breeders that carry the *Wx*^*b*^*, Wx*^*a*^ or *Wx*^*lv*^ alleles, respectively (Additional file 2: Figure S1). The typical *Wx*^*lv*^ allele of X55 harboured the same GT polymorphism as the *Wx*^*a*^ allele at the well-defined GT/TT polymorphism site that differentiates *Wx*^*a*^ and *Wx*^*b*^ alleles (Zhang et al. 2019). Initially, we planned to delete the entire 1st intron by choosing targeted sites of the 5′ and 3′ splicing sites of the 1st intron. However, we could not find proper target sequences and protospacer adjacent motifs (PAMs) in these two regions. Instead, we designed two target sites, Target1 and Target2, which were both located within the 1st intron but close to the 5′ and 3′ splicing sites of the *Wx* 1st intron, respectively (Fig. 1A). Target1’s PAM sequence (CCC) was located 33 bp away from the 5′splicing site of the *Wx* 1st intron, and Target2’s PAM sequence (CCA) was located 51 bp away from the 3′ splicing site of the *Wx* 1st intron. An edit vector pZZT477 (Fig. 1B, Additional file 3: Figure S2) expressing CRISPR/Cas9, two gRNAs (guide RNA, gRNA1 for Target1 and gRNA2 for Target2), and CP4/EPSPS that conferred glyphosate resistance selection marker was constructed and delivered into rice cells through *Agrobacterium*-mediated transformation. A total of 314 *glyphosate*-resistant transgenic plants were generated for these 4 rice inbred lines (Table 1). Our PCR assays on all 314 primary transformants detected 33 mutants with deletion of large DNA fragments (~ 1.0 kb) in the targeted intron region and deletion efficiencies ranging from 8.6–11.85% (Fig. 1C, Table 1). These 33 mutants included 12 KY131 mutants, 8 X32 mutants, 5 X35 mutants and 8 X55 mutants (Fig. 1C, Table 1).

**Table 1:** CRISPR/Cas9-mediated target deletion of Wx 1st intron

Inbred Name	No. of T_0_ plant tested	No. of 1st intron deleted lines (deletion efficiency, %)	No. of T_1_ lines tested
KY131	104	12 (11.5)	4
X32	68	8 (11.8)	4
X35	58	5 (8.6)	2
X55	84	8 (9.5)	4

We further investigated whether off-target effects occurred in our experiments. Using the online software CRISPR-P2.0 (http://crispr.hzau.edu.cn/CRISPR2/), we identified two potential off-target sites for both gRNAs used in this study (Additional file 1: Table S1). PCR amplification and DNA sequencing of predicted off-target sites were performed on both T_0_ mutants and some of their T_1_ offspring. As shown in Additional file 1: Table S1, no off-target effects were detected at the putative off-target loci in tested T_0_ plants their T_1_ offspring.

### Identification of Transgene-Free, Homozygous Mutants

T_1_ seeds of the primary transformants with a normal seed setting rate and other agronomic traits were selected for further screening of transgene-free, homozygous mutants. As shown in Table 1, 4, 4, 4 and 2 target mutant lines were obtained for KY131, X32, X55 and X35, respectively. Sequencing these 14 lines confirmed that ~ 1040 bp was deleted from the first intron (1128 bp in size) of their *Wx* genes. Further analyses indicated that these 14 mutants could be categorized into 3 mutant types according to differences in the number of nucleotides deleted. These three mutant types were named M1 (− 1041 bp), M2 (− 1042 bp), and M3 (− 1043 bp), and M2 was the predominant mutant (9/14) (Fig. 1D, Table 2). Given that the 5′ and 3′ intron splicing sites and an approximately 85-bp intron sequence were still retained within the 5′UTR of all mutants, a question arose regarding whether this short intron sequence in each mutant was still recognized as an intron and would be correctly spliced out from *Wx* transcripts. We conducted reverse transcriptase PCR (RT-PCR) to amplify fragments containing the 5′UTR and partial 5′ coding sequence of the *Wx* gene from cDNA samples derived from total mRNA of wild-type and mutant immature seeds. Sequencing these amplified fragments revealed that the remaining ~ 85-bp intron sequence was retained in mature mRNA of all mutants (in both *Wx*^*a*^ and *Wx*^*b*^ backgrounds) (Additional file 4: Figure S3), indicating that the remaining intron sequence was no longer recognized as an intron despite it still harbouring both splicing sites. Consequently, the 5′UTRs of M1, M2 and M3 mutants would have extra 87-, 86- and 85 bp sequences, respectively, compared to that of the wild type (Additional file 4: Figure S3). Since there was no extra translation start codon ATG found in these retained-intron sequences, we speculated that the translation initiation codon of mutant transcripts would be the same as that of wild-type transcripts.

**Table 2:** Mutation types and grain amylose contents (AC) of the first generation transgene-free, homozygous mutants

Background	Mutant line	No. of transgene-free plants tested	Mutation type	AC (%)
KY131	KY131	12	WT	13.8 ± 0.22d
	KY131-CR-1	8	M2	22.5 ± 0.07b
	KY131-CR-2	6	M2	22.7 ± 0.36b
	KY131-CR-3	12	M3	24.3 ± 0.12a
	KY131-CR-4	11	M2	21.5 ± 0.40c
X32	X32	12	WT	11.2 ± 0.06d
	X32-CR-1	8	M1	23.3 ± 0.37a
	X32-CR-2	8	M2	22.0 ± 0.65b
	X32-CR-3	12	M2	21.3 ± 0.34c
	X32-CR-4	9	M1	23.2 ± 0.25a
X55	X55	12	WT	28.1 ± 0.68a
	X55-CR-1	8	M2	28.1 ± 0.06a
	X55-CR-2	6	M2	28.4 ± 0.69a
	X55-CR-3	7	M2	28.3 ± 0.64a
	X55-CR-4	10	M2	27.9 ± 0.39a
X35	X35	12	WT	25.5 ± 0.68a
	X35-CR-1	9	M3	25.5 ± 0.43a
	X35-CR-2	12	M2	25.3 ± 0.34a

M1, M2, M3 represent mutants with 1041 bp, 1042 bp, 1043 bp deletion, respectively (Fig. 1D). AC values reported are mean ± SD. Different letters following the AC mean values stands indicate significant differences (*P* < 0.05)

### *Wx* Gene Expression was Significantly Increased in the KY131 and X32 Mutants

**Fig. 2:**
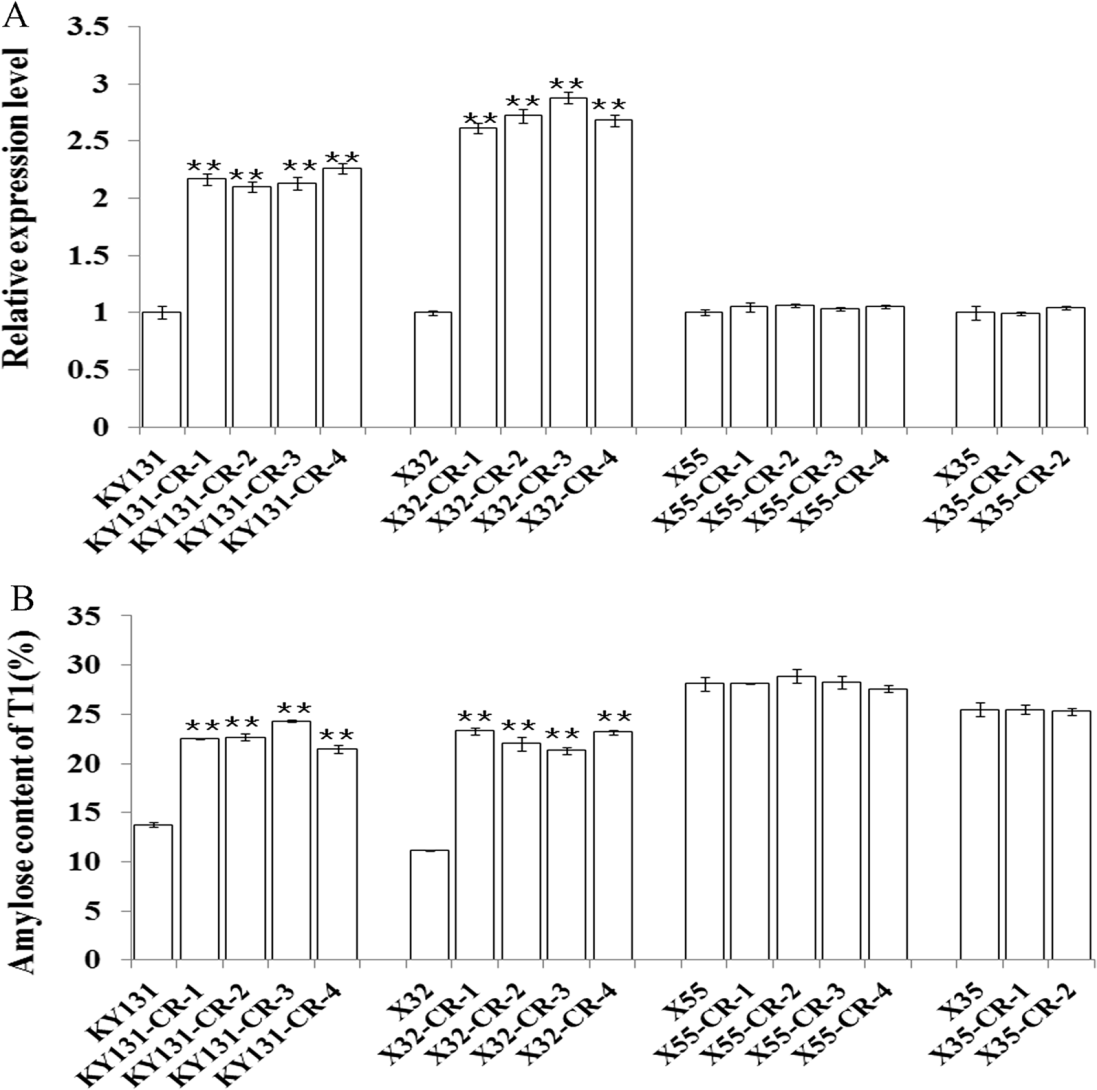
*Wx* gene expression and amylose content of endosperm in wild-type and T1 mutants. **A** Relative expression level of the *Wx* gene in wild-type and mutant 1st-generation transgene-free homologous plants. The expression noted in WT plants was arbitrarily set to 1, and the relative expression value of mutants was obtained by comparing the mutant expression to WT expression. **B** Amylose content of endosperm in wild-type and 1st-generation plants of transgene-free homologous mutants. Data are presented as the mean ± SD, **indicates a significant difference at *P* < 0.01 (t test)

To investigate the expression of the *Wx* gene in intron-deleted mutants, relative *Wx* gene mRNA levels in 10 DAP (days after pollination) seeds of different mutant plants and WT plants were detected by RT–qPCR. The results are shown in Fig. 2A. Compared to KY131 and X32 WT plants that both carried the *Wx*^*b*^ allele, the intron-deleted mutants of KY131 and X32 plants accumulated approximately 1–2 times more stable mRNA (Fig. 2A). Enhanced mRNA accumulation in mutants was highly correlated with increased AC in mutants. The greater the level of mature mRNA accumulation, the greater the AC (Table 2, Fig. 2), suggesting that removal of the first intron significantly affects *Wx* gene expression in a *Wx*^*b*^ background. However, the relative expression levels of the *Wx* gene between X35 and X55 WT plants (both have the *Wx*^*a*^ allele) and their intron-deleted mutants was minimal (Fig. 2A), demonstrating that removal of the first intron had a limited effect on *Wx* gene expression in a *Wx*^*a*^ background.

As mentioned above, the 5′UTRs of mutant M1, M2 and M3 mRNA were 87, 86 or 85 bp longer than that of WT (Additional file 4: Figure S3), and we wanted to know whether these differences influence *Wx* gene expression. Our *Wx* gene expression results and AC data demonstrated minimal change among the two X35 mutants, X35-CR-1 (M3), -CR-2 (M2), and WT X35 (Table 2, Figs. 1D, 2A), indicating that M2 and M3 mutant types had similar expression levels as WT. A similar notion could be derived from the comparison between four X55 mutants (all M2 mutant type) and WT X55 in terms of their *Wx* expression and AC (Table 2, Fig. 2). Thus, we concluded that the extra ~ 85/86 bp intron sequence retained in the 5′UTR of intron deletion mutants had minimal influence on *Wx* gene expression.

### Deletion of the *Wx* 1st Intron Substantially Increased the Grain Amylose Content of the KY131 and X32 Mutants

**Fig. 3:**
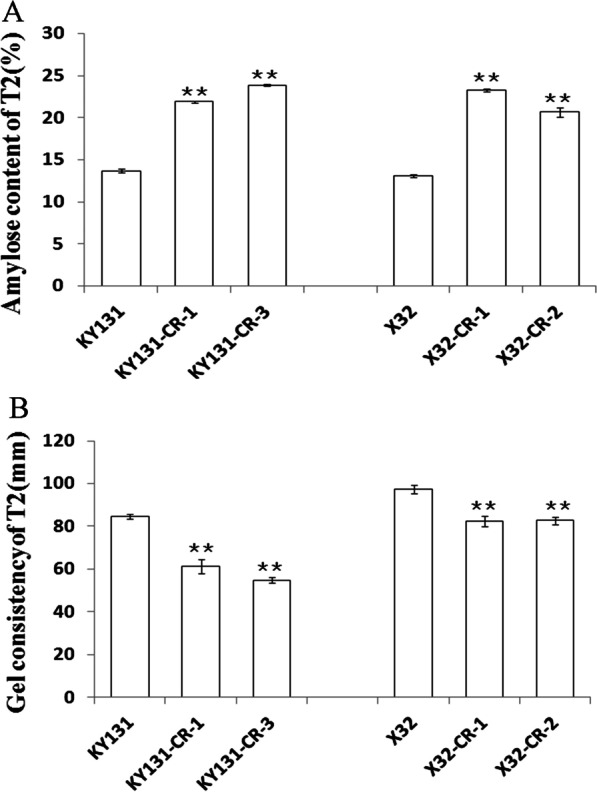
Amylose content and gel consistency of endosperm in wild-type and 2nd-generation mutant plants (T2). **A** Amylose content of endosperm in wild-type and 2nd-generation mutant plants (T2). **B** Gel consistency of endosperm in wild-type and 2nd-generation mutant plants (T2). Data are presented as the mean ± SD, **indicates a significant difference at *P* < 0.01 (t test)

The grain amylose content of transgene-free, homozygous mutants (first generation) and their corresponding WT plants was measured. The results are shown in Table 2. The amylose content of the KY131 and X32 mutants significantly increased from 13.0% to approximately 24.0%, whereas the amylose content of the X35 and X55 mutants was not significantly different (Table 2, Fig. 2B). These amylose content results were consistent with our *Wx* gene expression data. Significantly increased relative expression of the *Wx* gene was only observed in KY131 and X32 mutants that showed a significant AC increase but not in X35 and X55 mutants that showed similar ACs as their corresponding WT plants (Fig. 2A). We further analysed the grain amylose content of second-generation KY131 and X32 mutants. The results showed that the increased level of amylose content was consistent in both generations (Figs. 2B, 3A), suggesting that the AC change in mutants was genetically stable.

### Grain Quality Evaluation and Physicochemical Property Analysis of Mutant Grains

**Fig. 4:**
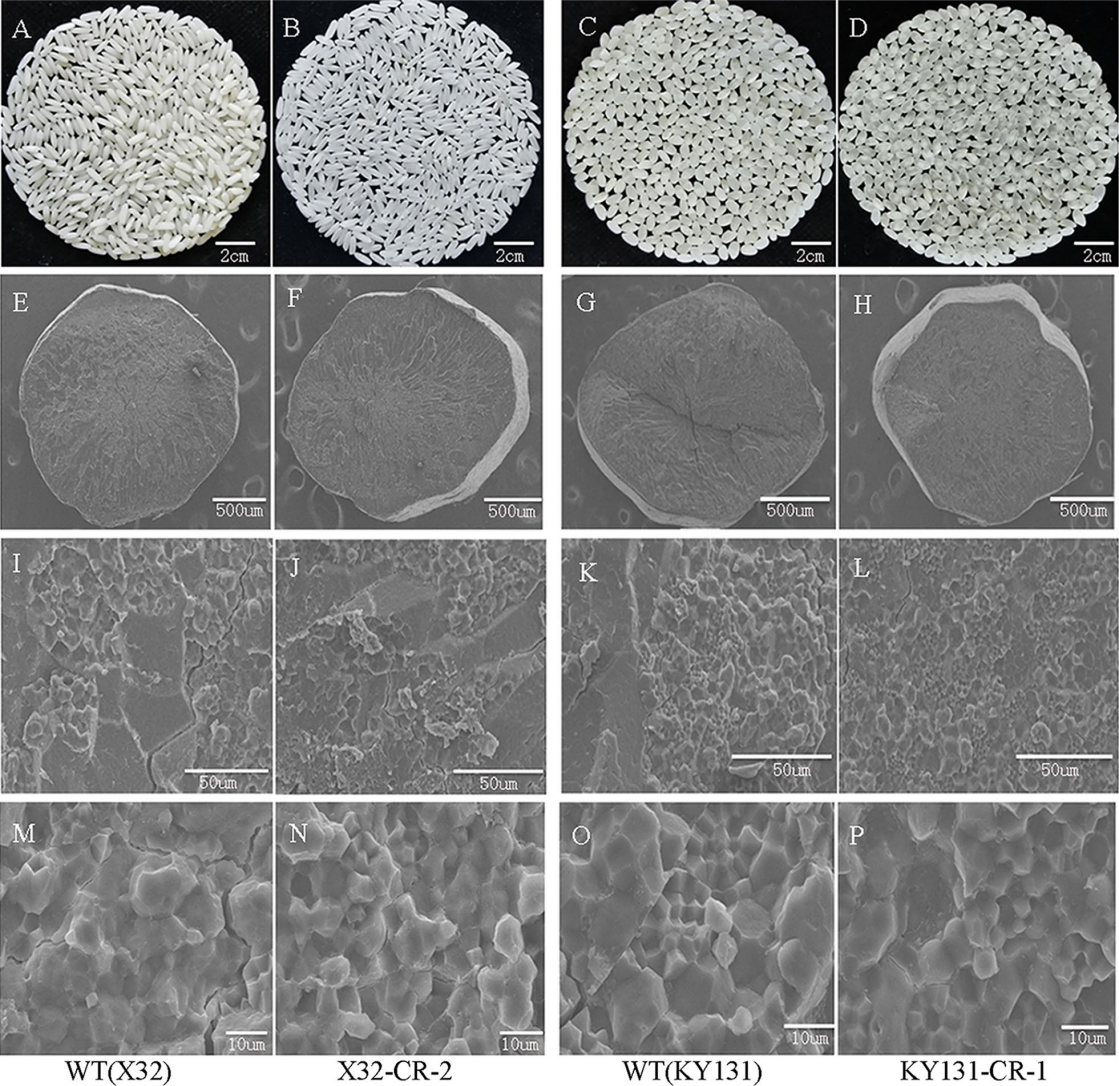
The appearance and morphology of starch granules of intron deleted mutants. **A**–**D** Milled rice from different mutants. **A** X32, **B** mutant X32-CR-2, **C** KY131, **D** mutant K131-CR-1. **E**–**P** SEM images showing the morphology of the starch granules. **E**, **I**, **M** were X32, **F**, **J**, **N** were mutant X32-CR-2, **G**, **K**, **O** were KY131and **H**, **L**, **P** were mutant K131-CR-1

It is well established that grain amylose content is closely linked to grain quality and affects grain physicochemical properties, such as GC and GT (Tian et al. 2009). To examine whether the deletion of the first intron of the *Wx* gene would affect other physicochemical properties of rice grains, we measured the physicochemical properties of rice grains derived from KY131-CR-1, KY131-CR-3, X32-CR-1, and X32-CR-2 mutant lines as well as the corresponding WT KY131 and X32 lines. Compared with grains of the WT plants, grains of 4 mutant plants showed no significant difference in gelatinization temperature/ASV (alkali spreading value) (Table 3). However, their amylose content increase slightly decreased the gel consistency of rice grains (Table 3), as expected. The RVA (Rapid Visco Analyzer) pasting property values also reflected similar results (Table 3, Fig. 3B). In addition, milling quality was also similar between the grains of KY131 and X32 WT plants and their mutants (Table 3). Grain transparency evaluation demonstrated that polished rice grains of X32 intron-deleted mutants had better transparency than those of X32 WT plants, indicating that appearance quality was improved in X32 intron-deleted mutants (Table 3, Fig. 4A, B). However, the improvement of appearance quality observed in X32 mutants was not found in KY131 intron-deleted mutants (Table 3, Fig. 4C, D). We further inspected whether amylose content changes influence the structure of starch granules by scanning electron microscopy (SEM) of transverse mature endosperm sections. Our results revealed no obvious difference in the morphology of starch granules from the endosperm between mutants and wild types (Fig. 4E–P).

**Table 3:** Appearance quality, milling quality and physiochemical properties of KY131 and X32 mutant lines (2nd generation of transgene-free homologous mutant plants)

Properties	KY131			X32		
	KY131(WT)	KY131-CR-1	KY131-CR-3	X32(WT)	X32-CR-1	X32-CR-2
Brown rice rate (%)	82.2 ± 0.14a	81.9 ± 0.08a	82.7 ± 0.02a	77.8 ± 1.2a	77.5 ± 0.77a	77.6 ± 0.52a
Polished rice rate (%)	70.6 ± 0.34a	70.2 ± 0.58a	70.7 ± 0.44a	65.2 ± 1.16a	66.6 ± 0.45a	65.1 ± 0.86a
Head rice rate (%)	66.8 ± 2.08a	65.4 ± 0.57a	64.5 ± 1.55a	59.7 ± 0.40a	54.0 ± 0.94b	54.8 ± 1.34b
Grain length (mm)	4.6 ± 0.09a	4.7 ± 0.01a	4.6 ± 0.06a	5.6 ± 0.11a	5.6 ± 0.02a	5.6 ± 0.01a
Grain width(mm)	2.7 ± 0.04a	2.6 ± 0.01a	2.7 ± 0.01a	2.0 ± 0.01a	1.9 ± 0.00a	1.9 ± 0.00a
Length/width ratio	1.6 ± 0.03a	1.8 ± 0.01a	1.7 ± 0.02a	2.8 ± 0.04a	3.0 ± 0.01a	3.0 ± 0.00a
Chalkiness rate (%)	10.0 ± 1.33a	9.1 ± 2.86a	10.9 ± 1.58a	14.7 ± 1.43a	8.8 ± 0.50b	9.7 ± 0.95b
Chalkiness degree (%)	2.9 ± 0.20a	3.7 ± 0.66a	3.6 ± 1.15a	4.9 ± 0.77a	2.8 ± 0.27b	3.4 ± 0.34b
Transparency grade	2 ± 0.00a	2 ± 0.00a	2 ± 0.00a	2 ± 0.00a	1 ± 0.00b	1 ± 0.00b
AC (%)	13.7 ± 0.08c	22.4 ± 0.19b	23.9 ± 0.44a	13.1 ± 0.12c	23.5 ± 0.34a	21.7 ± 0.41b
GC (mm)	84.6 ± 1.26a	61.2 ± 3.24b	54.8 ± 1.35c	97.4 ± 1.92a	82.4 ± 2.46b	82.7 ± 1.67b
ASV	6.0 ± 0.00c	6.9 ± 0.06a	6.5 ± 0.31b	1.3 ± 0.10a	1.2 ± 0.00a	1.2 ± 0.13a
PKV (RVU)	317.9 ± 8.38a	198.1 ± 8.79c	232.7 ± 2.63b	322.3 ± 1.93a	237.9 ± 3.35c	251.8 ± 5.82b
HPV (RVU)	176.4 ± 17.69a	154.4 ± 10.41a	172.6 ± 4.62a	131.9 ± 5.85b	143.7 ± 5.92ab	151.6 ± 2.53a
BDV (RVU)	141.5 ± 10.40a	43.7 ± 6.13b	60.0 ± 3.83b	190.4 ± 7.45a	94.2 ± 2.74b	100.2 ± 5.38b
CPV(RVU)	286.3 ± 20.19ab	260.0 ± 7.63b	298.4 ± 3.05a	202.4 ± 3.59b	270.3 ± 4.05a	274.3 ± 4.13a
SBV (RVU)	− 31.6 ± 12.76b	61.9 ± 1.34a	65.7 ± 1.56a	− 119.9 ± 5.5c	32.4 ± 0.70a	22.4 ± 1.80b
CSV (RVU)	109.9 ± 2.50b	105.6 ± 6.79b	125.7 ± 5.4a	70.5 ± 3.22b	126.6 ± 2.2a	122.7 ± 4.22a
PeT (Min)	6.1 ± 0.17b	6.6 ± 0.16a	6.4 ± 0.08ab	5.6 ± 0.08b	6.0 ± 0.07a	5.9 ± 0.12a
PaT (°C)	75.5 ± 0.44a	74.6 ± 0.89a	75.5 ± 0.43a	84.8 ± 0.03a	84.2 ± 0.98a	82.6 ± 0.46b

Values reported are mean ± SEM. Different letters following the mean values stands indicate significant differences (*P* < 0.05). AC, amylose content, GC, gel consistency, ASV, alkali spreading value; PV, peak viscosity; HPV, through viscosity or hot paste viscosity; CPV, final viscosity or cool paste viscosity; BDV, breakdown viscosity (BDV = PV-HPV); SBV, setback viscosity (SBV = CPV-PV); CSV, consistency viscosity (CSV = CPV-HPV); PeT, peak time; PaT, pasting temperature. All the viscosity parameters were expressed in rapid visco units (RVU)

## Discussion

The AC in rice endosperm is an important factor affecting rice ECQ (Tian et al. 2009). However, vast differences in regional consumer preference, market demand and functionality make ECQ difficult to define and standardize. In general, South Asians favour long, slender rice with a high AC and hard GC, whereas Southeast Asians prefer long grains with intermediate AC and soft GC. Such differences often hinder the spread of elite varieties to other countries with rice markets that demand rice with a different AC. Developing efficient, low-cost technology to modulate grain AC will provide a reliable solution to overcome this barrier. In addition, the development of specialized rice consumption creates more diversified demands for rice AC. For example, in recent years, the market has exhibited increasing demand for rice specialized for the rice wine industry and for patients with diabetes or high blood pressure (Sun et al. 2017). Therefore, it is of great commercial value to develop biotechnological methods to accurately adjust rice grain AC to satisfy diverse consumers.

With the rapid advance of CRISPR/Cas-mediated gene editing technologies (Gao 2021), researchers have exploited these novel powerful technologies for accurate modulation of rice grain AC. Studies on knocking out the *Wx* gene to produce glutinous rice (Ma et al. 2015; Xu et al. 2015; Zhang et al. 2018), fine-tuning *Wx* gene expression via regulatory promoter editing, and modulating the enzyme activity of GBSSI via base editing have recently been reported (Huang et al. 2020a, b, 2021; Xu et al. 2021; Zeng et al. 2020). In this study, we developed a new strategy to modify rice AC. We deleted the majority of the first intron of the rice *Wx* gene in both *Wx*^*a*^ and *Wx*^*b*^ backgrounds using CRISPR/Cas9 technology. Deletion of the first intron from inbred KY131 and X32 lines with a *Wx*^*b*^ allele significantly increased grain AC by greater than 10% (from 13.0% to approximately 24.0%) (Table 2, Fig. 2). However, such a phenomenon was not observed with X35 and X55 lines carrying a *Wx*^*a*^ allele. The grain AC of WT X35 and X55 and their intron-deleted mutants remained approximately similar (Table 2, Fig. 2). Our method will provide an efficient and rapid method to convert intermediate AC rice cultivars (mostly with *Wx*^*b*^ allele) to high AC cultivars, which facilitates elite rice cultivars of intermediate AC adapted to new plantation regions and consumer markets that favour high AC cultivars.

Deletion of the first intron generated a complete novel *Wx* allele which has other implications. First, removal of the first intron allowed us to gain insights into its regulation on *Wx* gene expression. Previous studies have showed that the GT/TT SNP at the 5′ splicing junction of the first intron is a major post-transcriptional regulation factor to affect rice grain AC (Cai et al. 1984; Isshiki et al. 1998; Samadder et al. 2008). By eliminating the splicing process of the first intron, we demonstrated that rice seeds accumulated approximately 1–2 times more stable mRNA, and their amylose content increased by approximately 10% (from 13.0% to approximately 24.0%) after removal of the first intron under the *Wx*^*b*^ background (Table 2, Fig. 2). In contrast, stable mRNA accumulation was noted, and seed AC remained approximately the same after the first intron was deleted under the *Wx*^*a*^ background (Table 2, Fig. 2). This finding provides a quantitative view of the extent to which the G to T SNP posttranscriptionally affects *Wx* gene expression. A previous study also demonstrated that the first intron of the rice *Wx* gene stimulates the expression of a foreign gene in rice protoplasts when it is placed in the 5′ UTR between the CaMV 35S promoter and GUS coding sequence, indicating that the first intron might act as a transcriptional enhancer (Li et al. 1995). This notion is plausible given that quite a few first introns with large sizes (~ 1.0 kb or more) and located within the 5′UTR of highly expressed genes, such as rice *Actin1*, maize *Ubi1* and *Adh1*, indeed function as transcriptional stimulators (Rose 2008, 2019). However, our data demonstrated that rice seeds carrying the *Wx*^*a*^ gene with or without the first intron accumulated approximately the same amount of stable mRNA and produced approximately the same amount of AC (Table 2, Fig. 2), strongly suggesting that the first intron of the rice *Wx* gene may not participate in transcriptional regulation. Of course, further well-designed studies are required to clarify this notion. Second, the new *Wx* allele without a first intron may help us in the future to further engineer new *Wx* alleles that could overcome the adverse effects of high temperature on rice grain quality. It has been well established that the majority of *japonica* rice strains carry the *Wx*^*b*^ allele and produce rice grains with low to intermediate AC, whereas the majority of *indica* rice strains harbour the *Wx*^*a*^ allele and produce rice grains with intermediate to high AC. In recent years, consumer preference has been increasingly changing to favour intermediate AC rice in the Chinese market, promoting the rapid spread of the *Wx*^*b*^ allele into *indica* rice cultivars. However, previous studies have noted that under elevated temperature, *Wx* gene expression is down-regulated in some *japonica* cultivars, resulting in less GBSSI protein production and leading to lower grain AC and lower grain quality. Presumably, the selection of the first intron 5′splicing site of the *Wx*^*b*^ gene is compromised under high temperatures (Larkin and Park 1999; Zhang et al. 2014a). The novel *Wx* allele created in this study may provide a promising solution to attenuate the adverse effect of high temperature on grain quality. Although removal of the first intron causes increased AC, further adjustment of the AC of the mutants with a deletion in the first intron to a suitable AC could be achievable using previously reported gene editing strategies (Huang et al. 2020a, 2021; Xu et al. 2021; Zeng et al. 2020). Engineering new *Wx* alleles that perform better under high temperature will be invaluable for rice production, particularly as global warming increasingly poses threats to global food security.

In conclusion, our study presented a novel and efficient strategy to modify rice grain AC, particularly to generate rice with a high AC from rice carrying a *Wx*^*b*^ allele. By deleting the first intron, we created a completely novel *Wx* allele. Further analyses of the first intron deletion mutants provided new insights into the regulation of *Wx* gene expression. The new *Wx* allele could serve as an excellent material for further engineering new *Wx* alleles that perform better under high temperatures and improve rice eating and cooking quality.

## Materials and Methods

### 1Plant Materials and Growth Conditions

The rice variety KY131 (KY131, *Wx*^*b*^ allele, *Oryza sativa* L. ssp. *japonica*) is an elite inbred variety broadly cultivated in the Northeast China region. X32 (*Wx*^*b*^ allele, *Oryza sativa* L. ssp. *indica*), X35 (*Wx*^*a*^ allele, *Oryza sativa* L. ssp. *indica*) and X55 (*Wx*^*lv*^ allele, *Oryza sativa* L. ssp. *indica*) are inbred lines provided by our breeders. The transgenic rice lines and transgene-free edited rice plants were all grown in a standard greenhouse (16-h light at 30 °C/8-h night at 22 °C) in the Life Science and Technology Center, China National Seed Group Co., LTD, Wuhan, China.

### Construction of the CRISPR/Cas9 Vector and Plant Transformation

Before constructing an editing vector, we employed the online software CRISPR-P2.0 (http://crispr.hzau.edu.cn/CRISPR2/) to identify proper editing target sites using the Nipponbare *Wx* gene (LOC_Os06g04200) as a reference. Two target sites located within the 1st intron but close to the 5′ or 3′ splicing site were selected (Fig. 1A). To confirm whether these two targeted sequences were suitable for editing the *Wx* gene of all 4 inbred lines, we amplified and sequenced fragments, including the targeted sequences and their flanking sequences from genomic DNA of all 4 inbred lines (KY31, X32, X35, and X55), using primer sets T1-F/T1-R (for Target1) and T2-F/T2-R (for Target2) (Additional file 1: Table S2). The CRISPR/Cas9 vector pZZT477 targeting the first intron of the *Wx* gene was constructed as previously described (Zhang et al. 2014b). The vector used in this study was based on the vector pCambia1300 backbone. The editing vector pZZT477 contained a Cas9 expression cassette driven by the sugarcane Ubi4 promoter, a *CP4–EPSPS* gene cassette (as a selection marker) driven by the CaMV 35S promoter, and two sgRNA expression cassettes driven by the rice U3 or U6 snRNA promoters (Fig. 1B, Additional file 3: Figure S2). The editing vector pZZT477 was transferred into *Agrobacterium tumefaciens* strain EHA105 by electroporation and consequently delivered into KY131, X32, X35 and X55 cells via* Agrobacterium*-mediated transformation as previously described (Hiei et al. 1997).

### Molecular Characterization of the Mutant Plants

Rice genomic DNA was extracted using a DNA Quick Plant System (TransGen Biotech, Beijing, China). Fifty nanograms of genomic DNA was used as a template to perform PCR amplification using Taq polymerase (Tiangen, Beijing, China). Genomic DNA of all transgenic herbicide-resistant T_0_ plants was first examined by PCR using the specific primers *CP4*-F/*CP4*-R to amplify the selection marker gene CP4/EPSPS. Only CP4 amplification-positive plants were selected for further editing status analysis using the primer set JC-F/JC-R (Additional file 1: Table S2). To examine the deletion details, PCR fragments amplified with the JC-F/JC-R primer set (Additional file 1: Table S2) from transgene-free homozygous plants were sequenced on an ABI3730XL capillary sequencer. To investigate the 5′UTR sequence of the mutant plant transcripts, we amplified and sequenced fragments using primers Transcript-F and Transcript-R (Additional file 1: Table S2) from first-strand cDNA samples derived from total RNA of immature seeds of intron-deleted mutants and wild-type plants. For off-target investigation, we identified two potential off-target sites (Additional file 1: Table S1) using the online software CRISPR-P2.0 (http://crispr.hzau.edu.cn/CRISPR2/). PCR amplification of fragments flanking the two potentials was performed with primer pairs Off-T1-F/R and Off-T2-F/R. Amplified fragments were consequently sequenced and analysed.

### Selection of Transgene-Free Homozygous Mutants

To select transgene-free T_1_ plants, the rice leaves of T_1_ transgenic plants from selected lines were sampled for GMO testing. An Applied Biosystems 7900HT instrument was used to conduct quantitative real-time PCR for detecting transgene residues. To ensure that there were no transgenic residues in selected homozygous mutants, we used 11 primer pairs to amplify T-DNA components, including the *CaMV35S* promoter (CaMV35S-F/R), *SsUbi4* promoter (P#SsUbi4-F/R), *NOS* terminator (T#NOS-F/R, NOS-F/R), *OCS* terminator (T#OCS-F/R), *CaMV35S* terminator (T#35SpolyA-F/R), *Cas9* (CAS9 Os-F/R), *Epsps-CP4* (CAS9 Os-F/R), and vector backbone region (pCAMBIA1301-1-vector-F/R, pCAMBIA1301-2-vector-F/R, and pCAMBIA1301-3-vector-F/R). Only homologous mutants with PCR products negative for all 11 primer pairs were selected for further analysis. All 11 primer pairs used for GMO tests are listed in Additional file 1: Table S2.

### RNA Extraction and RT–PCR Analysis

Total RNA was extracted from grains after 10 days of grain filling using TRIzol reagent (Invitrogen). First-strand cDNA was synthesized using the Transcriptor First Strand cDNA Synthesis Kit (Roche). Quantitative RT–PCR analysis was performed using 2 × SYBR Green PCR Master Mix on an Applied Biosystems 7900HT (Applied Biosystems). The rice *Actin1* gene was used as an internal control for normalization. Sequences of primers used in qPCR, Wx-RT-F/R and OsActin7-F/R, are listed in Additional file 1: Table S2.

### Scanning Electron Microscopy of Starch Granules

Rice grains were dried in an oven at 42 °C for 2 days and cooled in a desiccator. Cross-sections of the samples were manually snapped and sputter-coated with gold palladium on copper studs. Magnifications of 500 × and 2000 × were used to observe endosperm and starch granule morphology.

### Evaluation of Rice Quality

The rice quality was measured following the procedure described in GB/T 15683-2008 and NY/T 83-2017, and each sample was tested thrice. RVA pasting properties were detected with a RVA according to the manufacturer’s instructions (NewPort Sci. Co., Australia).

### Statistical Analysis

The data were analysed by using Student’s unpaired t test in Microsoft Excel. Differences were considered to be significant at *P* < 0.05 or *P* < 0.01.

## Supplementary Information

**Additional file 1: Table S1.** Analysis of potential off-target effects. **Table S2.** Primers used in this study.

**Additional file 2: Figure S1.** The *Wx* genotypic sequences of the four rice inbred used in the test. The two SNPs defining *Wx*^*b*^, *Wx*^*a*^ and *Wx*^*lv*^ alleles were shown in red. Int1-1: the first neucleotide of the 1st intron of the *Wx* gene; Ex10-115: the 115th neucleotide of the 10th exon of the *Wx* gene.

**Additional file 3: Figure S2.** The structure diagram of vector pZZT477.

**Additional file 4: Figure S3.** The 5′UTR sequences of the matured mRNAs of the *Wx* gene of different mutants. Sequences were derived from PCR amplification of DNA fragments covering the 5′UTR and a partial coding sequence from total RNA isolated from mutant and wild type immature seeds. Primer Transcript-F and Transcript-R (Table S2) were used to perform in PCR amplification. The extra sequence presented in each mutant type were marked in black. The translation start codon ATG were marked in red. The G/T polymorphism site that differentiates *Wx*^*a*^ and *Wx*^*b*^ alleles was marked in different colours.

## Data Availability

All data generated or analyzed during this study are included in this published article and its Additional files 1–4. The materials used and/or analyzed during the current study are available from the corresponding authors on reasonable request.

## Abbreviations

AC: Amylose content
bp: Base pairs
CRISPR: Clustered regularly interspaced palindromic repeats
*Cas*: CRISPR-associated protein
ECQ: Eating and cooking quality
EPSPS: 5-Enolpyruvylshikimate-3-phosphate synthase
GBSSI: Granule-bound starch synthase I
GC: Gel consistency
GT: Gelatinization temperature
gRNA: Guide RNA
GUS: *β*-Glucoronidase
PAM: Protospacer adjacent motif
qRT-PCR: Quantitative reverse transcriptase-PCR polymerase chain reaction
RVA: Rapid visco analyzer
SEM: Scanning electron microscopy
SNP: Single nucleotide polymorphism
UTR: Untranslated region
WT: Wild type
*Wx*: Waxy
