# Supplementary material for: Targeted Deletion of the First Intron of the Wxb Allele via CRISPR/Cas9 Significantly Increases Grain Amylose Content in Rice
Source: Rice (N Y). 2022 Jan 4;15:1. doi: 10.1186/s12284-021-00548-y (PMC8727654; doi:10.1186/s12284-021-00548-y)
Supplement: Supplementary file 1 — Additional file 1: Table S1. Analysis of potential off-target effects. Table S2. Primers used in this study. [file 12284_2021_548_MOESM1_ESM.docx]

**Targeted deletion of the first intron** **of the** *Wx^b^* **allele *via* CRISPR/Cas9 significantly increases grain amylose content in rice**

Xingdan Liu^1^, Qi Ding^2,3^, Wenshu Wang^2^, Yanling Pan^2^, Chao Tan^2,^, Yingbo Qiu^2^, Ya Chen^2^, Hongjing Li^2^, Yinlong Li^2^, Naizhong Ye^2^, Nan Xu^2^, Xiao Wu^2,3^, Rongjian Ye^2,3^, Jianfeng Liu^1,2,3^*, Chonglie Ma^2,3,^*

1. College of Agronomy, Hunan Agricultural University, Changsha 410128, China
2. Life Science and Technology Center, China National Seed Group Co., LTD, Wuhan, Hubei 430206, China
3. State Key Laboratory of Crop Breeding Technology Inovation and Integration, China National Seed Group Co., LTD, Wuhan, Hubei 430206, China

* Corresponding Authors , C. Ma (machonglie@gxaas.net), J. Liu ( [liujianfeng@hunau.edu.cn](mailto:liujianfeng@hunau.edu.cn) )

**Supplementary information**

**Table S1 Analysis of potential off-target effects.**

| Target | Putative off-target   locus | Sequence of the putative off-target site | No. of  mismatching   bases | No. of plants  sequenced | No. of plants with  mutations | |
| --- | --- | --- | --- | --- | --- | --- |
| Target1 | Chr01:+39756906 | GAGATCAGTCTCAATAAGAG | 4 | 90 | | 0 |
| Target2 | Chr04:-24553085 | TCGCCTAAAAGAATTAGTAC | 4 | 90 | | 0 |

Mismatched bases are shown in red

**Table S2 Primers used in this study.**

| **Primer sets** | **Sequence (5′ To 3′)** |
| --- | --- |
| T1-F | ggaccgggtaaaatgtgttgcg |
| T1-R | tcagctagcccaccatcttgtgg |
| T2-F | ctagccacccaagaaactgctcc |
| T2-R | gacgaacacgacgttcatgcc |
| JC-F | tcacgcaacggcgctacaaatagc |
| JC-R | gtttgtgtgtgcttacagccatgg |
| CaMV35S-F | cgacagtggtcccaaaga |
| CaMV35S-R | aagacgtggttggaacgtcttc |
| P#SsUbi4-F | ccctcctcccccgttataaa |
| P#SsUbi4-R | gactgggatttggatggatga |
| T#NOS-F | gattagagtcccgcaattatacatttaa |
| T#NOS-R | ttatcctagtttgcgcgctatattt |
| NOS-F | atcgttcaaacatttggca |
| NOS-R | attgcgggactctaatcata |
| T#35SpolyA-F | tgtgtgagtagttcccagataagg |
| T#35SpolyA-R | ggtttcttatatgctcaacacatga |
| CAS9 Os-F | caagcagtccggcaagac |
| CAS9 Os-R | ggacacctgggccttctg |
| CP4-F | aagtgcagccaccagaggaaac |
| CP4-R | caagctcacgggccaagtg |
| pCAMBIA1301-1-vector-F | atagcatcggtaacatgagcaaagtctg |
| pCAMBIA1301-1-vector-R | tcggcacaaaatcaccactcgatac |
| pCAMBIA1301-2-vector-F | atgtcgcacaagtcctaagttacg |
| pCAMBIA1301-2-vector-R | ctccggttctagtcgcaagtattc |
| pCAMBIA1301-3-vector-F | cgagctgctatctgaatacatcgc |
| pCAMBIA1301-3-vector-R | gtcggtgcctggttgttcttg |
| T#OCS-F | tccgctctaccgaaagttacg |
| T#OCS-R | tcaaggtttgacctgcacttc |
| OsActin7-F | gctgaccgtatgagcaaggagatc |
| OsActin7-R | gctaagggaggcaaggatgga |
| Wx-RT-F | cgacaggaaaatcccactgatc |
| Wx-RT-R | tccagtacccagaagaacgatc |
| Transcript-F | catcaggaagaacatctgcaag |
| Transcript-R | cccaagcgtccttgtactggtc |
| Wx-1-F | ccctagccacccaagaaactg |
| Wx-1-R | ctccagtgtcaggtccgtagat |
| Wx-2-F | agacaggtacgagagggtgagg |
| Wx-2-R | tggcggtgatgtacttgtcc |
| Wx-3-F | accctgcactactgtccatcg |
| Wx-3-R | atgtggaaaccagtcttgcctt |
| Wx-4-F | gccgcttgctcatctcatc |
| Wx-4-R | cacacccagaagagtacaacatca |
| Off-T1-F | ctccacggttccagttgttt |
| Off-T1-R | tggtcctctgtgcacttttg |
| Off-T2-F | tgtcccaacccagattcttc |
| Off-T2-R | aatggcatccccaaatgtta |

**Figure S1 The *Wx* genotypic sequences of the four rice inbred used in the test.** The two SNPs defining *Wx^b^*, *Wx^a^* and *Wx^lv^* alleles were shown in red. Int1-1: the first neucleotide of the 1^st^ intron of the *Wx* gene; Ex10-115: the 115^th^ neucleotide of the 10^th^ exon of the *Wx* gene.

**Figure S2 The structure diagram of vector pZZT477.**

**Figure S3 The 5’UTR sequences of the matured mRNAs of the *Wx* gene of different mutants.** Sequences were derived from PCR amplification of DNA fragments covering the 5`UTR and a partial coding sequence from total RNA isolated from mutant and wild type immature seeds. Primer Transcript-F and Transcript-R (Table S2) were used to perform in PCR amplification. The extra sequence presented in each mutant type were marked in black. The translation start codon ATG were marked in red. The G/T polymorphism site that differentiates *Wx^a^* and *Wx^b^* alleles was marked in different colours.
