# Supplementary figures and images for: Targeted Deletion of the First Intron of the Wxb Allele via CRISPR/Cas9 Significantly Increases Grain Amylose Content in Rice
Source: Rice (N Y). 2022 Jan 4;15:1. doi: 10.1186/s12284-021-00548-y (PMC8727654; doi:10.1186/s12284-021-00548-y)

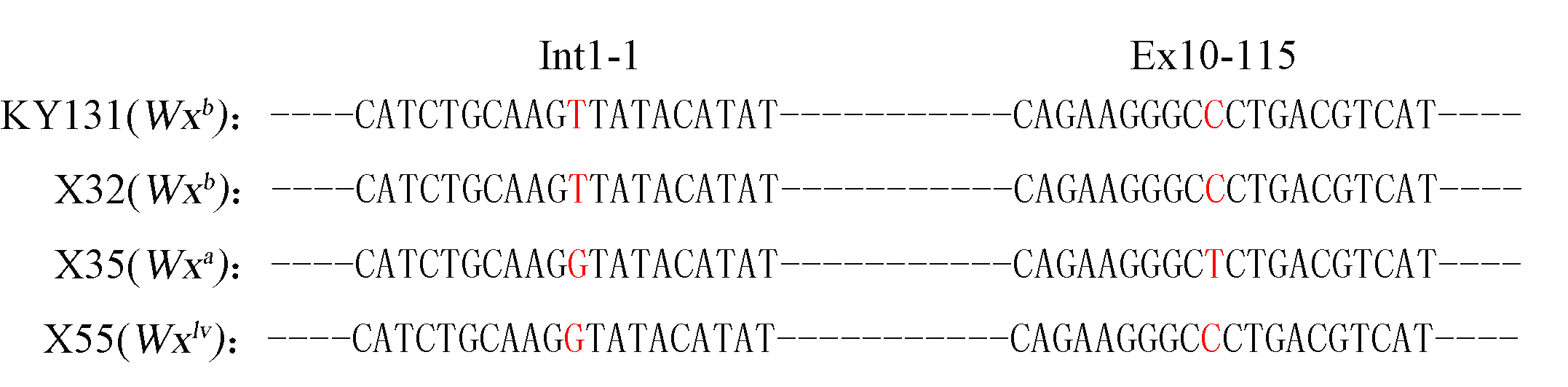

Supplement: Supplementary file 2 — Additional file 2: Figure S1. The Wx genotypic sequences of the four rice inbred used in the test. The two SNPs defining Wxb, Wxa and Wxlv alleles were shown in red. Int1-1: the first neucleotide of the 1st intron of the Wx gene; Ex10-115: the 115th neucleotide of the 10th exon of the Wx gene. [file 12284_2021_548_MOESM2_ESM.tif]

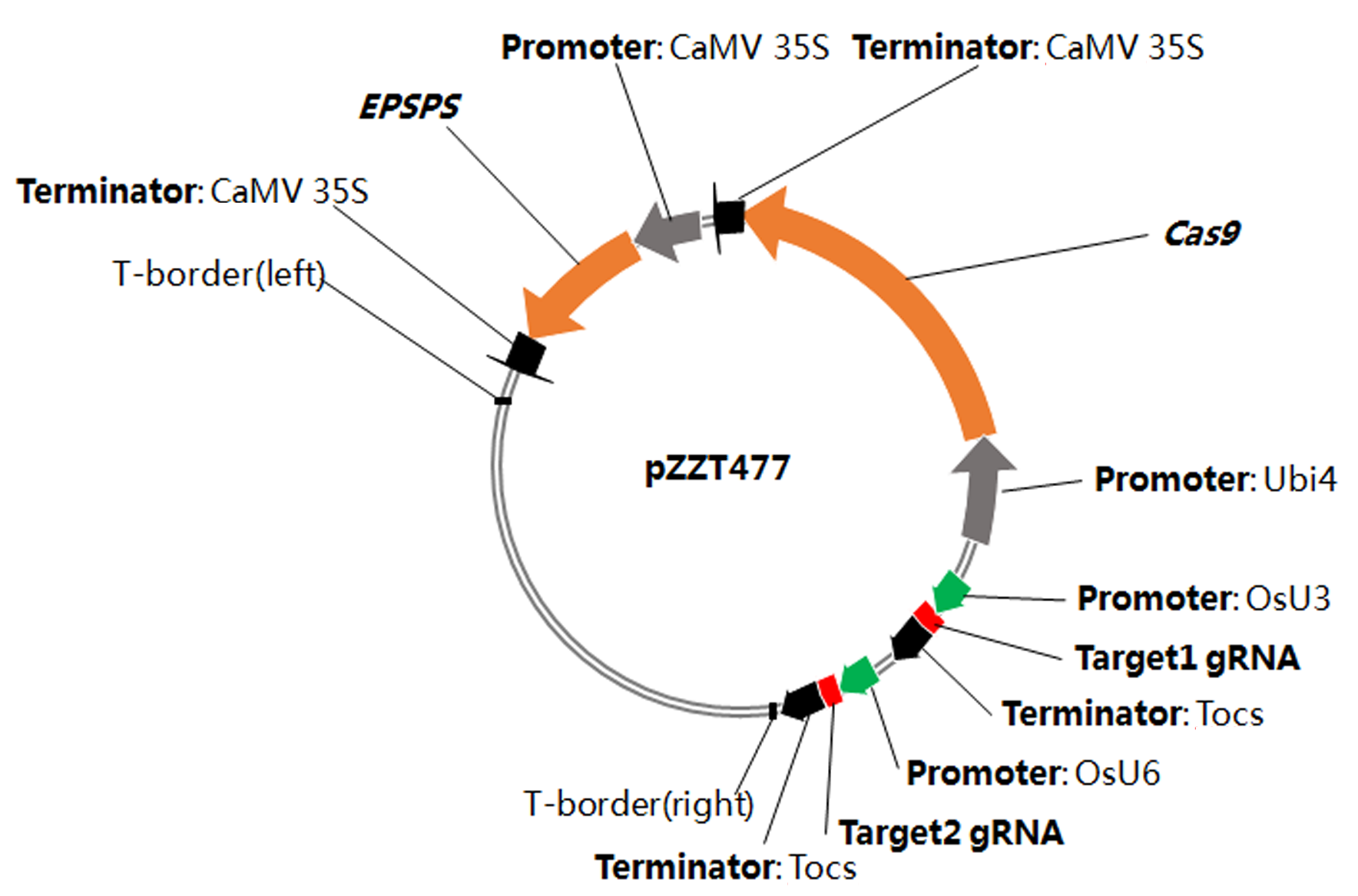

Supplement: Supplementary file 3 — Additional file 3: Figure S2. The structure diagram of vector pZZT477. [file 12284_2021_548_MOESM3_ESM.tif]

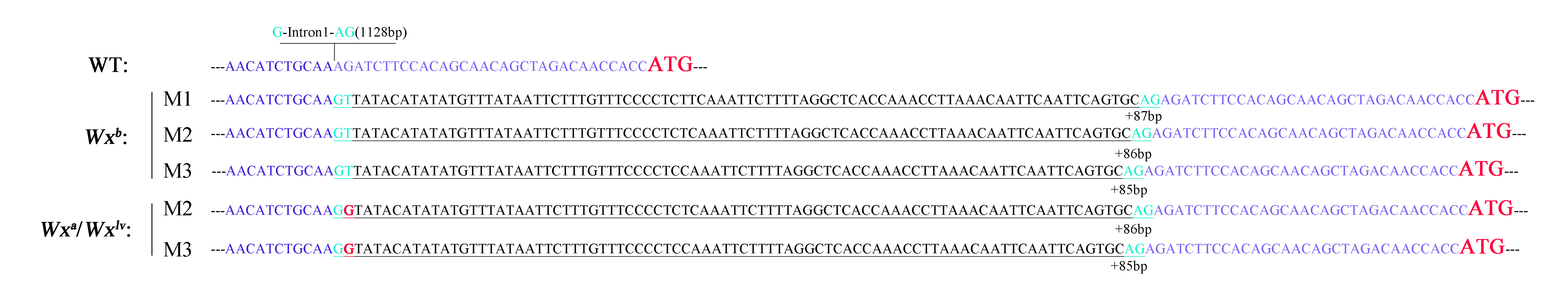

Supplement: Supplementary file 4 — Additional file 4: Figure S3. The 5′UTR sequences of the matured mRNAs of the Wx gene of different mutants. Sequences were derived from PCR amplification of DNA fragments covering the 5′UTR and a partial coding sequence from total RNA isolated from mutant and wild type immature seeds. Primer Transcript-F and Transcript-R (Table S2) were used to perform in PCR amplification. The extra sequence presented in each mutant type were marked in black. The translation start codon ATG were marked in red. The G/T polymorphism site that differentiates Wxa and Wxb alleles was marked in different colours. [file 12284_2021_548_MOESM4_ESM.tif]
